# Crude Oil Prices Forecast Based on Mixed-Frequency Deep Learning Approach and Intelligent Optimization Algorithm

**DOI:** 10.3390/e26050358

**Published:** 2024-04-24

**Authors:** Wanbo Lu, Zhaojie Huang

**Affiliations:** 1 School of Management Science and Engineering, Southwestern University of Finance and Economics, Chengdu 611130, China; 2 School of Statistics, Southwestern University of Finance and Economics, Chengdu 611130, China; huangzhaojie@smail.swufe.edu.cn

**Keywords:** crude oil prices forecast, decomposition–integration paradigm, mixed-frequency deep learning, sparrow search algorithm, fuzzy entropy

## Abstract

Precisely forecasting the price of crude oil is challenging due to its fundamental properties of nonlinearity, volatility, and stochasticity. This paper introduces a novel hybrid model, namely, the KV-MFSCBA-G model, within the decomposition–integration paradigm. It combines the mixed-frequency convolutional neural network–bidirectional long short-term memory network-attention mechanism (MFCBA) and generalized autoregressive conditional heteroskedasticity (GARCH) models. The MFCBA and GARCH models are employed to respectively forecast the low-frequency and high-frequency components decomposed through variational mode decomposition optimized by Kullback–Leibler divergence (KL-VMD). The classification of these components is performed using the fuzzy entropy (FE) algorithm. Therefore, this model can fully exploit the advantages of deep learning networks in fitting nonlinearities and traditional econometric models in capturing volatilities. Furthermore, the intelligent optimization algorithm and the low-frequency economic variable are introduced to improve forecasting performance. Specifically, the sparrow search algorithm (SSA) is employed to determine the optimal parameter combination of the MFCBA model, which is incorporated with monthly global economic conditions (GECON) data. The empirical findings of West Texas Intermediate (WTI) and Brent crude oil indicate that the proposed approach outperforms other models in evaluation indicators and statistical tests and has good robustness. This model can assist investors and market regulators in making decisions.

## 1. Introduction

Energy is crucial for promoting national prosperity, improving well-being, and ensuring social stability. The use of energy has caused the transformation of human production technology and significantly promoted the development level of productivity. As global industrialization continues, crude oil, a critical nonrenewable energy product, has become an essential elemental energy, chemical feedstock, and strategic resource, affecting world stability, national economic development, and enterprise decisions.

Crude oil exhibits generic commodity qualities as well as financial and political features [1]. Its price volatility can be attributed to various economic and noneconomic factors, further compounded by the collective impact of market and nonmarket forces. Previous research revealed that crude oil prices are influenced by economic variables, including production, consumption, settlement currency, alternative energy prices, and global economic conditions indexes [2,3,4]; financial market factors, such as financial market trading characteristics, international hot money speculation, and exchange rate changes; and other nonmarket factors, such as geopolitical conflicts, extreme climate change, and energy technology progress [5,6]. International crude oil price forecasting is an essential issue of the economic research of energy. The diversity and complexity of the influencing factors significantly increase the difficulty of accurate forecasting.

International crude oil prices have experienced significant fluctuations over the past 20 years, characterized by rapid rises and sharp falls, shortened boom cycles, and fewer smooth transition periods [7]. In 2001, the Internet bubble burst, and the global economy slowed down. From September to November, the price of Brent crude oil fell from USD 29.43/barrel to USD 17.68/barrel, with a maximum decline of 40%. The financial crisis of 2008 collapsed the prices from USD 140/barrel in June to approximately USD 33/barrel in December, a decline of more than 76% in 6 months. Crude oil prices declined and bottomed out in early 2016 owing to the shale oil revolution. Oil prices plunged by 42% in 2018 as the United States (US)–China trade war escalated, and oil producers continued to increase production in response to the supply gap caused by US sanctions on Iran. In 2020, with the global new coronavirus outbreak, the global energy demand fell by approximately 4.5%, and oil demand fell by an unprecedented 9.3%. As the market panic spread, the settlement price of West Texas Intermediate (WTI) crude oil futures in May was USD −37.63/barrel, closing negative for the first time in history. After 2021, the widespread use of COVID-19 vaccines and government economic stimulus measures drove up the energy demand and global energy prices. International oil prices increased by over 60% in 2021.

As economic globalization deepens, energy plays an increasingly pivotal role in supporting economic development and social stability. The accurate forecasting of crude oil prices is crucial for market analysts, investors, policymakers, and enterprises. This forecasting not only offers valuable insights into market dynamics but also informs the formulation of energy policies and guides investment decisions and strategies. Therefore, developing a scientific and more accurate method for predicting crude oil prices is imperative. Early methods employed for crude oil price forecasting primarily relied on econometric models. With the rapid development of artificial intelligence and big data methods, machine learning techniques have been widely used. In recent years, scholars have found that the decomposition–integration technique can further enhance the accuracy and robustness of prediction by decomposing complex nonlinear, high-volatility, and irregular time series data into multiple sub-series that are easier to process and predict, and then predicting these sub-series separately and finally integrating the results. For the components derived from the decomposition, existing studies have applied and refined various machine learning methods for forecasting. However, only a few studies have combined deep learning methods with traditional econometric models, but in fact, each of them has its own advantages and disadvantages [8]. Traditional econometric models often possess strong explanatory power. For instance, the generalized autoregressive conditional heteroskedasticity (GARCH) model effectively captures heteroskedasticity in time-series data, aiding in understanding the mechanisms behind fluctuations in crude oil prices. However, these models are constrained by their reliance on assumptions such as residual normal distribution and linear relationships. In contrast, deep learning networks excel at handling large-scale data and complex nonlinear relationships. Nevertheless, their internal mechanisms are challenging to interpret, and they exhibit sensitivity to hyperparameters while being difficult to optimize. In addition, most studies have only used past price information for future prediction, ignoring the influence of low-frequency economic variables. Actually, the introduction of monthly economic variables will bring about the issue of mixed-frequency forecasting.

Therefore, under the decomposition–integration paradigm, following the principle of data-driven modeling [9,10], we construct a novel hybrid model to predict crude oil prices accurately, which combines mixed-frequency deep learning approaches, the traditional econometric model, and the intelligent optimization algorithm. This study seeks to leverage the combined methods to fully exploit their respective strengths, thereby effectively enhancing the predictive accuracy of the model.

The primary contributions of the paper are as follows:

(1) In this paper, the deep learning approach and GARCH model are integrated to accurately predict the low-frequency and high-frequency mode components derived from decomposition. Thus, the proposed model effectively combines the strengths of deep learning and traditional econometric models, demonstrating superior predictive accuracy compared to other models. The convolutional neural network–bidirectional long short-term memory network-attention mechanism (CBA) model has a long-term memory capability, effectively illustrating the bidirectional characteristics and multilevel saliency factors, and is a good fit for nonlinear series [11]. Furthermore, the GARCH model can well portray short-term volatility clustering [12].
(2) In this paper, the idea of mixed-frequency (MF) prediction is incorporated into the deep learning method, and then the mixed-frequency long short-term memory (MFLSTM) and MFCBA models are constructed to predict each low-frequency component. Incorporating the monthly low-frequency global economic conditions (GECON) index into the deep learning model by the mixed-data sampling (MIDAS) technique can significantly enhance forecast accuracy.
(3) In this paper, Kullback–Leibler (KL) divergence is used to determine the optimal combination of the variational mode decomposition (VMD) method’s number of decomposition layers K and penalty factor α rather than relying on subjective judgment, resulting in a more efficient and robust decomposition and improved prediction performance. In addition, whether the LSTM and CBA parameters are reasonably chosen significantly impacts the prediction accuracy. Therefore, the sparrow search algorithm (SSA) is applied to determine the best parameter combination for the LSTM, MFLSTM, and MFCBA deep learning models. The intelligent optimization algorithm SSA, chosen for the proposed model, converges faster than the alternatives, and the prediction accuracy of the model optimized using SSA surpasses others.

The rest of the sections are arranged in the following way: Section 2 conducts a literature review of relevant studies. Section 3 introduces the VMD algorithm optimized by KL divergence (KL-VMD), the fuzzy entropy (FE) algorithm, the CBA deep learning model, the SSA, and the novel KL-VMD-MF-SSA-CBA-GARCH (KV-MFSCBA-G) model framework. In Section 4, we conduct empirical analyses, model comparisons, and statistical tests. Section 5 is the discussion where we compare the model with existing models in the literature, compare SSA with other optimization algorithms, and analyze the economic significance and future directions of this study. Section 6 concludes the paper.

## 2. Literature Review

Oil prices have complex properties, such as nonlinearity and dynamics, making accurate forecasting tricky. Over the years, numerous researchers have investigated various modeling methods to increase the precision of oil price forecasts. The proposed methods primarily include traditional econometric models, machine learning methods, hybrid models under the decomposition–integration paradigm, and econophysics approaches.

Traditional econometric models construct estimating equations based on economic theory. They can recognize variables influencing crude oil prices efficiently and produce interpretable findings, including the random walk model [13], generalized exponential predictor model [14], autoregressive (AR) model [2], autoregressive integrated moving average (ARIMA) model [15,16], GARCH model [12], GARCH-MIDAS model [4], vector autoregressive (VAR) model [17], hidden Markov model [18], error correction model [19,20], etc. It is worth mentioning that Mohammadi and Su (2010) and Xiang (2022) have both validated the usefulness of the ARIMA-GARCH combination model for modeling and forecasting the conditional mean and volatility of international oil prices, especially in short-term forecasting [21,22].

In general, linear and stationary time series are assumed in applying such models. However, crude oil prices typically do not satisfy these conditions. Therefore, traditional models might face challenges in effectively capturing oil prices’ complex and nonlinear characteristics.

With the continuous progress of artificial intelligence, data mining, and other emerging technologies, machine learning models, including support vector machines (SVM), extreme learning machines (ELM), extreme gradient boosting (XGBoost), neural networks, and random forest, have become practical tools to cope with the characteristics of sequence randomness, nonlinearity, multi-noise, and dynamic changes, and have been popularly adopted in oil price forecasting. For example, Xie et al. (2006) [23] used SVM for oil price forecasting and found that its predictive accuracy outperformed that of ARIMA and back-propagation neural network models. Moshiri and Foroutan (2006) [24] compared the prediction ability of the artificial neural network (ANN) with the ARIMA and GARCH models and concluded that the ANN approach exhibits enhanced forecasting performance for crude oil prices. Mingming and Jinliang (2012), Gumus and Kiran (2017), Tang et al. (2018), and Wang et al. (2018) [25,26,27,28] constructed multi-wavelet recurrent neural network, XGBoost, random vector functional link network, and ELM, respectively. They all attained high prediction accuracy in oil price forecasting. Karasu and Altan (2022) [29] proposed a model incorporating LSTM, technical indicators, and the chaotic Henry gas solubility optimization technique, which can cope well with crude oil prices’ chaotic and nonlinear characteristics.

Most machine learning methods using oil price data for iterative learning and training can accurately portray the nonlinearity of time series, leading to enhanced prediction accuracy. However, these methods also encounter challenges, such as reduced interpretability of the prediction results, a tendency to fall into local minima, parameter sensitivity, and overfitting [7,30].

Therefore, to further enhance forecasting accuracy, many studies have employed hybrid models within the decomposition–integration framework for oil price forecasting, with most achieving satisfactory results. The decomposition–integration technique decomposes crude oil prices into distinct subsequences, each characterized by a relatively simple structure. Subsequently, econometric or machine learning models are applied to predict each subsequence. Finally, the predicted value is derived by adding the forecast values of each component [31]. For example, Fang et al. (2023) [30] forecasted Brent crude oil spot prices using a feedforward neural network (FNN) after decomposition using empirical mode decomposition (EMD) with the slope-based method. Wu et al. (2019) [32] combined the ensemble EMD (EEMD) method and LSTM to predict international oil prices, revealing that the model has a broad application. Jiang et al. (2022) [33] combined the EEMD and gated recurrent units (GRU) with the seagull optimization algorithm (SOA) for forecasting. Huang and Deng (2021) [34] used the improved signal-energy rule to optimize VMD, and the moving window (MW) method was applied to propose the VMD-LSTM-MW method, which is confirmed to be superior for the oil price empirical results. Zhao et al. (2021) [35] applied VMD optimized by particle swarm optimization (PSO) to decompose crude oil prices. Subsequently, they adopted the ARMA model to predict the smooth series and the SVM model to predict the unsmooth series. They empirically verified that the combined model surpassed others in accuracy and robustness. Jovanovic et al. (2022) and T. Li et al. (2021) [36,37] both used VMD and then used LSTM and random sparse Bayesian learning, respectively, to make crude oil price predictions. The research findings illustrate that their models outperform several other approaches for various evaluation indicators.

Additionally, some recent studies have adopted non-traditional approaches, such as the use of econophysics. For instance, Leng and Li (2020) [38] employed Bayesian and econophysical methods to analyze the dynamic prediction of crude oil prices. Gharib et al. (2021) [39] utilized econophysics techniques to detect bubbles and predict the collapse time of oil prices. Aslam et al. (2022) [40] employed robust econophysics-based multifractal detrended cross-correlation analysis to examine the nonlinear structure of the interconnection between geopolitical risks and the energy market. Li et al. (2022) [41] investigated the risk resonance effect between crude oil prices and the Chinese stock market using econophysics, asset pricing theory, and machine learning.

Combining the literature, we find that although the hybrid forecasting models within the decomposition–integration framework have exhibited commendable forecasting capabilities, there remains ample scope for enhancement:

(1) Many studies have proven the VMD algorithm’s role in modeling crude oil prices [34,35,36,37]. Nevertheless, VMD requires the pre-specification of K and α before decomposition. Therefore, the objective selection of the parameters for VMD deserves attention.
(2) For each component obtained after decomposition, studies typically explore and develop diverse machine learning methods for prediction. Recently, some studies have also explored quadratic decomposition for highly complex components or residual terms, followed by modeling using machine learning techniques to enhance prediction accuracy [5,7,42,43]. However, deep learning approaches and econometric models each have their own strengths and weaknesses. Few studies have investigated the combination of them to predict components with different frequency characteristics.
(3) A large proportion of these studies rely primarily on historical crude oil price data for future predictions, ignoring the potential impact of exogenous variables. However, monthly economic indicators might affect oil price dynamics, such as the growth of the global economy, which will increase the overall demand in the global commodity market, significantly increasing prices [3].

Thus, we construct the KL-VMD-MF-SSA-CBA-GARCH (KV-MFSCBA-G) model within the decomposition–integration paradigm. This model begins with decomposing crude oil prices into components with different frequency characteristics using VMD optimized by KL divergence. Subsequently, the FE algorithm is applied to identify and classify the component frequency characteristics into low-frequency and high-frequency terms. Then, MFSCBA is employed to forecast low-frequency trend terms combined with relatively low-frequency macroeconomic variable data, while the GARCH model is applied to forecast high-frequency disturbance terms. The SSA is used to optimize the parameter combination of deep learning models, which partially mitigates the challenge of parameter tuning difficulty. Lastly, the final forecasts for WTI and Brent are derived by aggregating the predictions of each component.

## 3. Methodology

This section mainly explains the KL-VMD method, FE algorithm, CBA deep learning model, SSA, and the procedures for building the KV-MFSCBA-G framework.

### 3.1. Variational Mode Decomposition Optimized by Kullback–Leibler Divergence

VMD is an enhancement based on the EMD introduced by Dragomiretskiy and Zosso in 2014 [44]. The EMD can recognize complicated signal properties with no previous knowledge. However, end-point effects and mode component aliasing restrict its decomposition performance. Therefore, scholars have proposed the VMD algorithm to compensate for these limitations. Unlike the recursive decomposition of EMD, VMD uses a variational decomposition—essentially, multiple adaptive Wiener filters. This transformation facilitates adaptive segmentation of the signal components, improving noise robustness and attenuating the end-point effect.

The primary goal of VMD is to establish and determine the following variational problem:

(1) $$\underset{\left\{u_{k}\right\},\left\{\omega_{k}\right\}}{\min}\left\{\sum_{k=1}^{K}{\left\|\partial_{t}\left[\left(\delta \left(t\right)+\frac{j}{\pi t}\right)*u_{k}\left(t\right)\right]e^{-j\omega_{k}t}\right\|}_{2}^{2}\right\},$$

(2) $$s.t.\sum_{k=1}^{K}u_{k}=f,$$

where f represents the signal, K denotes the number of modes, $u_{k}$ is the kth mode component, and $\omega_{k}$ is the kth frequency center. $\delta \left(t\right)$ is the Dirac function, and ∗ is the convolution operator.

By imposing the quadratic penalty function and Lagrange multiplier λ, the restricted variational issue is converted into an unconstrained variational task, as seen below:

(3) $$L\left(\left\{u_{k}\right\},\left\{\omega_{k}\right\},\lambda \right)=\alpha \sum_{k=1}^{K}{\left\|\partial_{t}\left[\left(\delta \left(t\right)+\frac{j}{\pi t}\right)*u_{k}\left(t\right)\right]e^{-j\omega_{k}t}\right\|}_{2}^{2}\;+{\left\|f\left(t\right)-\sum_{k=1}^{K}u_{k}\left(t\right)\right\|}_{2}^{2}+\left\langle \lambda \left(t\right),f\left(t\right)-\sum_{k=1}^{K}u_{k}\left(t\right)\right\rangle ,$$

where α is the quadratic penalty factor used to decrease the disturbance from Gaussian noise.

The alternating direction method of multipliers, Parseval’s theorem, and Fourier isometric transform can be applied to solve Equation (3). After alternate optimization iterations, the expressions for $u_{k},w_{k}$, and λ are as follows:

(4) $${\hat{u}}_{k}^{n+1}\left(\omega \right)=\frac{\hat{f}\left(\omega \right)-\sum_{i\neq k}{\hat{u}}_{i}\left(\omega \right)+\frac{\hat{\lambda }\left(\omega \right)}{2}}{1+2\alpha {\left(\omega -\omega_{k}\right)}^{2}},$$

(5) $$\omega_{k}^{n+1}=\frac{\int_{0}^{\infty }\omega {\left|{\hat{u}}_{k}^{n+1}\left(\omega \right)\right|}^{2}d\omega }{\int_{0}^{\infty }{\left|{\hat{u}}_{k}^{n+1}\left(\omega \right)\right|}^{2}d\omega },$$

(6) $${\hat{\lambda }}^{n+1}\left(\omega \right)={\hat{\lambda }}^{n}\left(\omega \right)+\gamma \left(\hat{f}\left(\omega \right)-\sum_{k}{\hat{u}}_{k}^{n+1}\left(\omega \right)\right),$$

where γ is the noise tolerance, and n is the number of iterations. ${\hat{u}}_{k}^{n+1}\left(\omega \right),\;{\hat{u}}_{i}\left(\omega \right),\;\hat{f}\left(\omega \right)$, and $\hat{\lambda }\left(\omega \right)$ are the Fourier transforms of $u_{k}^{n+1}\left(t\right),\;u_{i}\left(t\right),\;f(t)$, and λ(t), respectively.

The procedure for the iterative of VMD is as follows:

(1) Initialize ${\hat{u}}_{k}^{1},\omega_{k}^{1},\lambda^{1}$, and N, n=0.
(2) Use Equations (4) and (5) to update ${\hat{u}}_{k}$ and $\omega_{k}$.
(3) Update $\hat{\lambda }$ using Equation (6).
(4) Assume that the accuracy convergence criterion is ε>0; if it does not satisfy $\sum_{k}{\left\|{\hat{u}}_{k}^{n+1}-{\hat{u}}_{k}^{n}\right\|}_{2}^{2}/{\left\|{\hat{u}}_{k}^{n}\right\|}_{2}^{2}<\varepsilon$ and n<N, then revert to step (2). Otherwise, end the iteration and print the last ${\hat{u}}_{k}$ and $\omega_{k}$.

Although VMD overcomes the drawbacks of the traditional EMD and its improved methods, the values of K and α must be set before the decomposition. The selection of parameters can significantly influence the decomposition results. Therefore, determining the optimal parameter combination [K,α] is imperative. We apply the *KL* divergence (relative entropy) to optimize VMD’s K value and penalty factor α [45,46].

The *KL* divergence is the degree of similarity between two probability distributions P and Q, which is thus calculated as:

(7) $$D_{KL}\left(P∥Q\right)=\sum_{i=1}^{N}P\left(x_{i}\right)\log\frac{P\left(x_{i}\right)}{Q\left(x_{i}\right)},$$

where $P(x_{i})$ represents the probability distribution of the actual data, and $Q(x_{i})$ denotes the distribution predicted by the model. A decrease in the *KL* divergence indicates a higher degree of alignment between the estimated probability distribution and the actual probability distribution.

When determining the optimal parameters for VMD, the range for K is set between 3 and 10, and the range for α is set between 100 and 2500 with a step size of 100. The optimal combination is identified by selecting the values of K and α corresponding to the minimum relative entropy.

### 3.2. Fuzzy Entropy

Scholars have introduced diverse entropy measures to assess the disorder and complexity features of time series, including approximate and sample entropies. However, both methodologies define vector similarity using the unit step function. In reality, the boundary between modes is frequently ambiguous. Seeking to improve sample entropy, Chen et al. 2007 [47] proposed an FE method based on the fuzzy theory, which uses the affiliation function to compute the fuzzy similarity between various hidden modes. Specifically, for a given time series $x\left(t\right),t=1,2,\ldots ,T$, its FE is calculated as follows.

(1) Sequence segmentation.

Based on time series x(t), construct the embedding vector X(i); the embedding dimension is m.

(8) $$X_{i}^{m}=\left[x\left(i\right),x\left(i+1\right),\ldots ,x\left(i+m-1\right)\right],\;1\leq i\leq T-m+1.$$

(2) Calculate the distance.

The distance of two vectors X(i) and X(j), $d_{ij}^{m}$, is the Chebyshev distance.

(9) $$d_{ij}^{m}=d\left[X_{i}^{m},X_{j}^{m}\right]=\underset{k=0,1,\ldots ,m-1}{\max}\left\{\left|\left[x\left(i+k\right)-x_{0}\left(i\right)\right]-\left[x\left(j+k\right)-x_{0}\left(j\right)\right]\right|\right\},$$

where $x_{0}(i)=\left[\sum_{j=0}^{m-1}x(i+j)\right]/m$ is the baseline vector of X(i).

(3) Calculate the similarity.

The similarity $D_{ij}^{m}$ between X(i) and X(j) is estimated based on fuzzy affiliation functions with parameters n and r.

(10) $$D_{ij}^{m}\left(n,r\right)=u\left(d_{ij}^{m},n,r\right),$$

where $u(d_{ij}^{m},n,r)$ is the fuzzy affiliation function.

(4) Define the function $\varphi^{m}(n,r)$.

Calculate the value of $\varphi^{m}(n,r)$ based on $D_{ij}^{m}$.

(11) $$\varphi^{m}=\frac{1}{T-m+1}\sum_{i=1}^{T-m+1}\left(\frac{1}{T-m}\sum_{j=1,i\neq j}^{T-m+1}D_{ij}^{m}\right).$$

Similarly, construct the vector $X_{i}^{m+1}$ and compute $\varphi^{m+1}$.

(12) $$\varphi^{m+1}\left(n,r\right)=\frac{1}{T-m}\sum_{i=1}^{T-m}\left(\frac{1}{T-m-1}\sum_{j=1,i\neq j}^{T-m}D_{ij}^{m+1}\right).$$

(5) Calculate the *FE* of $x\left(t\right)$.

(13) $$FE\left(m,n,r,T\right)=\ln\varphi^{m}\left(n,r\right)-\ln\varphi^{m+1}\left(n,r\right).$$

Equation (13) shows that the *FE* is affected by the parameters m, n, r, and T. The embedding dimension, denoted as m, is generally taken as m=2. n determines the gradient of the similarity tolerance threshold, and a larger  n corresponds to a steeper gradient. Chen et al. [47] recommend using a smaller integer value, such as 2 or 3, when calculating the gradient. r is the similarity tolerance threshold typically set within the range of $0.1\sigma_{sd}\sim 0.25\sigma_{sd}$. T is the sample length.

### 3.3. CNN-BiLSTM-Attention Deep Learning Model

#### 3.3.1. Convolutional Neural Network (CNN)

An FNN with convolutional, pooling, and fully connected layers constitutes the CNN. Convolutional layers are central and use kernels for convolutional computation and feature generation. Pooling layers perform secondary subsampling to prevent overfitting, and fully connected layers at the end of the model integrate features for the final output. This paper applies 1D-CNN to price data for efficient local feature extraction.

#### 3.3.2. Bidirectional Long Short-Term Memory Network (BiLSTM)

LSTM is a unique RNN introduced by Hochreiter and Schmidhuber in 1997 [48]. LSTM can learn long-term dependence information, thus solving the gradient vanishing or exploding problem, and is more applicable to predict time series with complex nonlinear and stochastic features.

At time t, the following formulas can be used to determine the forgetting gate ($f_{t}$), input gate ($i_{t}$), output gate ($o_{t}$), candidate memory unit (${\tilde{C}}_{t}$), memory cell ($C_{t}$), and the hidden state ($h_{t}$) of LSTM:

(14) $$\left\{\begin{array}{l} \;\;f_{t}=\sigma \left(W_{f}\cdot \left[h_{t-1},x_{t}\right]+b_{f}\right),\\ \;\;i_{t}=\sigma \left(W_{i}\cdot \left[h_{t-1},x_{t}\right]+b_{i}\right),\\ {\;\;o}_{t}=\sigma \left(W_{o}\cdot \left[h_{t-1},x_{t}\right]+b_{o}\right),\\ \;\;{\tilde{C}}_{t}=\tanh\left(W_{c}\cdot \left[h_{t-1},x_{t}\right]+b_{c}\right),\\ \;\;C_{t}=f_{t}⊙C_{t-1}+i_{t}⊙{\tilde{C}}_{t},\\ \;{\;h}_{t}=o_{t}⊙\tanh\left(C_{t}\right), \end{array}\right.$$

where $x_{t}$ represents the input value at time t, σ is the Sigmoid function with a range of (0,1), tanh(·) is a hyperbolic tangent function with a range of (−1,1), ⊙ represents the Hadamard product. ${W_{f},W_{i},W}_{o},\;W_{c}$ and $b_{f},\;b_{i},b_{o},\;b_{c}$ are the weight and bias parameters of the forgetting gate, input gate, output gate, and memory cell, respectively.

BiLSTM is an improvement of *LSTM*, stacking the forward and backward-propagating *LSTM* layers, creating a bidirectional recurrent structure. It can fully consider past and future information, effectively capture interaction characteristics within data, and has been applied in crude oil price prediction [49]. Therefore, we employ BiLSTM to extract time series features.

BiLSTM’s combination of hidden layer states is described as follows:

(15) $$\left\{\begin{array}{l} \;\;h_{f}=LSTM\left(x_{t},h_{f-1}\right),\\ \;\;h_{b}=LSTM\left(x_{t},h_{b-1}\right),\\ \;\;h_{t}=W_{f}h_{f}+W_{b}h_{b}+c_{t}, \end{array}\right.$$

where LSTM denotes the *LSTM* unit operation process. $x_{t}$ represents the input. ${h_{f},h}_{b},h_{f-1}$, and $h_{b-1}$ are the hidden states for the forward and backward propagation of cells at the current and previous time, respectively. $W_{f}$ and $W_{b}$ are the weights of the forward and backpropagating units, respectively. $c_{t}$ is the bias optimization parameter.

#### 3.3.3. Attention Mechanism (Attention)

Due to the extensive extraction of temporal and spatial features, this paper optimizes the model using the attention mechanism, which concentrates on crucial information by allocating distinct weights to input characteristics. The prioritization of vital information is evident in the weight calculation process, where higher importance causes greater assigned weights. The computation process is outlined below:

(1) Calculate the correlation vector.

Suppose the output of the BiLSTM layer is $h_{1},h_{2},...,h_{t},...,h_{T},t\in [1,T]$, and input them to the attention layer. The similarity score $S_{t}(h_{t})$ for each moment is calculated by the tanh function as follows:

(16) $$S_{t}\left(h_{t}\right)=\tanh\left(W_{h}h_{t}+b_{h}\right),$$

where $S_{t}(h_{t})$ represents the degree of correlation between the state $h_{t}$ and the output state. $W_{h}$ and $b_{h}$ are the weight and bias, respectively.

(2) Conduct attention scoring.

The attention weight $a_{t}$ of the hidden layer unit $h_{t}$ is obtained from the softmax function as follows:

(17) $$a_{t}=softmax\left(\frac{\exp\left(S_{t}\right)}{\sum_{t=1}^{T}\exp\left(S_{t}\right)}\right),$$

where $a_{t}$ denotes the importance of the state, and softmax⁡(·) is the activation function.

(3) Obtain the output.

To obtain the output C optimized by the attention mechanism, each $h_{t}$ is multiplied by its corresponding $a_{t}$ and summed as seen below:

(18) $$C=\sum_{t=1}^{T}a_{t}h_{t}.$$

#### 3.3.4. CNN-BiLSTM-Attention

**Figure 1:**
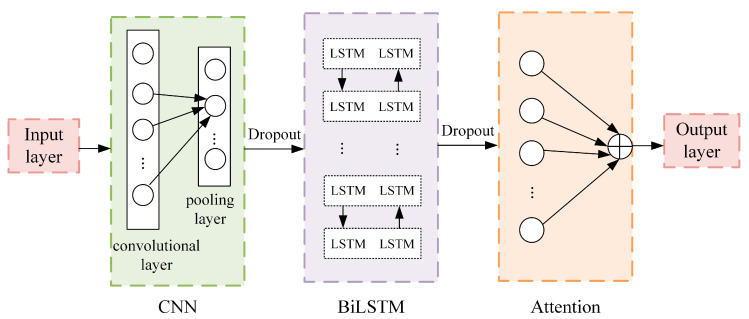
CNN-BiLSTM-Attention deep learning model.

A CNN layer, BiLSTM layer, and attention mechanism comprise the CBA model (Figure 1). The steps of the method are described below:

(1) The CNN layer consists of convolutional, pooling, and dropout layers, and is designed to capture spatial features from the input data.
(2) BiLSTM is then trained based on the local features obtained from the CNN layer to learn the patterns of internal dynamic variation and obtain the forward and reverse time series temporal features.
(3) The extracted temporal and spatial features are fed into the attention mechanism, enhancing the model’s attention to crucial features during learning and improving prediction accuracy.

### 3.4. Sparrow Search Algorithm

The SSA is an intelligent optimization algorithm introduced by Xue J and Shen B in 2020 [50], which searches for the best solution by simulating the sparrow’s foraging process. This algorithm is relatively innovative and has strengths in efficiently finding optimal solutions and demonstrating rapid convergence. The optimization process of the SSA is outlined below:

(1) The discoverer position $X_{i,j}^{t+1}$ is updated using the following formula:

(19) $$X_{i,j}^{t+1}=f\left(x\right)=\left\{\begin{array}{l} \;\;X_{i,j}^{t}exp\left[-\frac{i}{\alpha \cdot {iter}_{max}}\right],\;\;\;\;\;\;R_{2}<S_{T},\\ \;\;X_{i,j}^{t}+QL,\;\;\;\;\;\;\;\;\;\;\;\;\;\;\;\;\;\;\;\;\;R_{2}\geq S_{T}, \end{array}\right.$$

where $X_{i,j}$ represents the position of the ith sparrow in dimension j. $R_{2}$ and $S_{T}$ are the warning and safety values, respectively.

(2) The joiner position $X_{i,j}^{t+1}$ is updated using the following formula:

(20) $$X_{i,j}^{t+1}=\left\{\begin{array}{c} \;\;\mathrm{Qexp}\left[\frac{X_{worst}-X_{i,j}^{t}}{i^{2}}\right],\;\;\;\;\;\;\;\;\;\;\;\;\;\;\;\;\;\;\;\;i>\frac{n}{2},\\ \;\;X_{P}^{t+1}+\left|X_{i,j}^{t}-X_{P}^{t+1}\right|A^{+}L,\;\;\;\;\;\;otherwise, \end{array}\right.$$

where $X_{p}$ and $X_{worst}$ are the optimal and worst positions, respectively.

(3) Suppose that 10–20% of the sparrows in the flock are alert to the threat. Those who are aware of the danger will promptly relocate to a safe zone. The position of their vigilantes $X_{i,j}^{t+1}$ can be expressed as seen below:

(21) $$X_{i,j}^{t+1}=\left\{\begin{array}{l} \;\;X_{best}^{t}+\beta \left|X_{i,j}^{t}-X_{best}^{t}\right|,\;\;\;\;\;\;\;\;\;\;\;\;f_{i}>f_{g},\\ \;\;X_{i,j}^{t}+K\left(\frac{\left|X_{i,j}^{t}-X_{worst}^{t}\right|}{\left(f_{i}-f_{w}\right)+\varepsilon }\right),\;\;\;\;\;\;\;\;\;\;f_{i}=f_{g}, \end{array}\right.$$

where $X_{best}$ represents the current global optimal position. $f_{i},f_{g}$, and $f_{w}$ are the fitness values of the current individual sparrow, the global optimal, and the global worst, respectively.

The following are the steps of SSA:

(1) Set the initial value of the population, the ratio of predators and joiners, and the number of iterations.
(2) After computing the fitness values, sort them in descending order.
(3) Apply Equation (19) to update the discoverer position.
(4) Apply Equation (20) to update the joiner position.
(5) Apply Equation (21) to update the vigilante position.
(6) Compute the fitness value and update the sparrow positions.
(7) Evaluate if the stop criterion is met. If so, quit and print the result; otherwise, repeat steps (2)–(6).

### 3.5. KL-VMD-MF-SSA-CBA-GARCH Model

**Figure 2:**
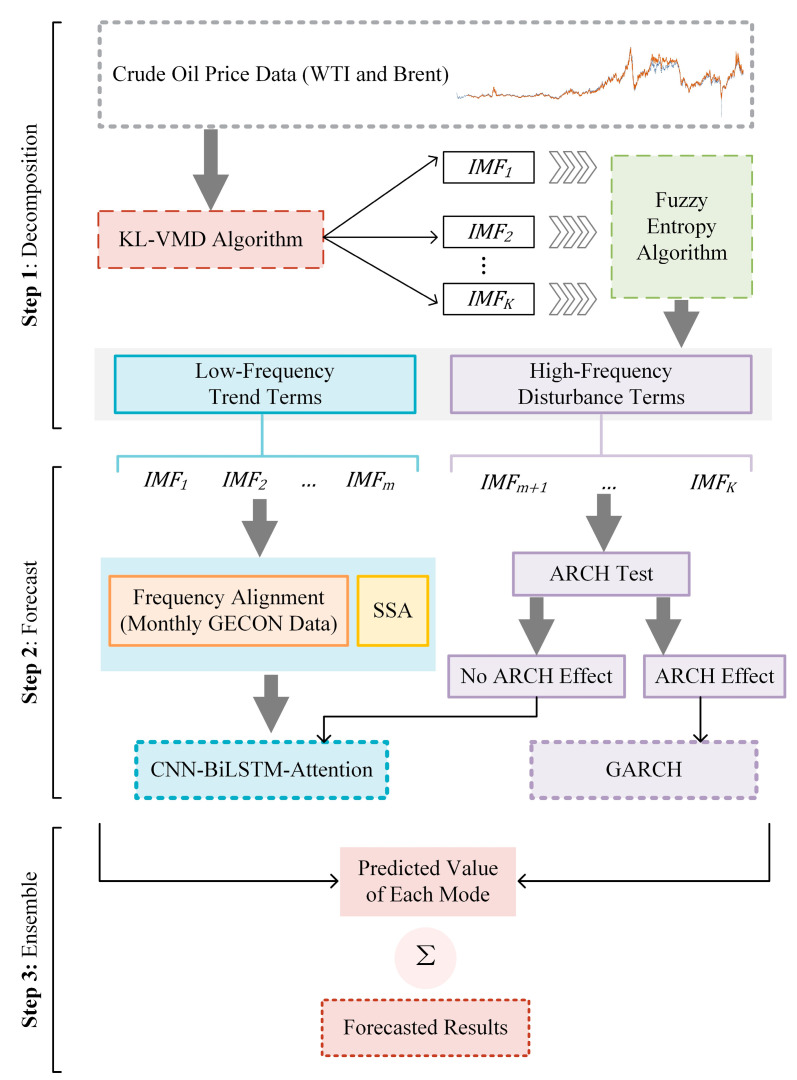
The process of the KL-VMD-MF-SSA-CBA-GARCH model.

In this paper, we constructed a nonlinear mixed-frequency decomposition–integration approach to forecast crude oil prices, namely, the KV-MFSCBA-G model. This model combines the KL-VMD method, FE algorithm, mixed-frequency prediction idea, CNN, BiLSTM, attention mechanism, SSA, and GARCH model. Deep learning methods and traditional econometric models are selected for modeling in response to components with different frequency characteristics. Mixed-frequency data (low-frequency macroeconomic data) are effectively combined for prediction in deep learning networks. Furthermore, we employ SSA to optimize the parameter combination of CBA. Three steps and details of the model are presented in Figure 2.

#### 3.5.1. Step 1: Price Decomposition and Characteristic Recognition

The primary objective is to decompose the raw data to derive several components. The KL-VMD method can effectively decompose international crude oil prices into K mode components with distinct frequency characteristics. These components are independent, have a straightforward structure, and demonstrate robust regularity. The mode components can be categorized into two groups according to the frequency characteristics.

(1) **Low-frequency trend terms**

The low-frequency trend terms, marked by a small amplitude, reflect external environmental influences on crude oil price changes. This contributes to long-term stable price trends and sometimes can be significantly impacted by major events, causing rapid value shifts.

(2) **High-frequency disturbance terms**

The high-frequency disturbance terms have random short-period variations in crude oil prices caused by transient factors. Despite its frequent short-term changes, it lacks long-term impact. In addition, volatility clustering is observed, where significant price swings correspond to sharp fluctuations, and smaller price changes coincide with minor fluctuations.

The analysis above indicates that different mode components have distinct frequency characteristics. Adopting particular prediction models for components with different frequency features and incorporating mixed-frequency exogenous explanatory variable data can enhance prediction accuracy. We use the *FE* complexity algorithm to classify the frequency characteristics of the mode components into two categories. The *FE* algorithm quantifies the disorder within the dynamic state of the time series, with a higher computed *FE* value indicating increased complexity in the time series. Therefore, we classify the mode components based on the *FE* value in this paper. First, we calculate the complexity of the mode component *k* based on the *FE* algorithm, denoted as ${FE}_{k},\;k=1,2,\ldots ,K$. Second, we set the critical value λ (0.05). Finally, we classify each mode component IMF based on the FE value. ${IMF}_{1},\;{IMF}_{2},...,\;{IMF}_{m}$ are recognized as low-frequency trend terms (FE<λ), and ${IMF}_{m+1},...,\;{IMF}_{K}$ are recognized as high-frequency disturbance terms (FE>λ).

#### 3.5.2. Step 2: Mode Components Forecasting Combined with Mixed-Frequency Data

Different prediction methods are used for different mode component categories, using MFSCBA to forecast low-frequency trend terms and GARCH to forecast high-frequency disturbance terms. Furthermore, we introduce monthly macroeconomic variables for the low-frequency trend terms to enhance the prediction accuracy. Nevertheless, owing to the mismatch in frequency between the introduced exogenous explanatory variables (monthly) and the forecast target (daily), it is imperative to consider mixed-frequency data forecasting. The modeling process is outlined below.

(1) **Forecasting low-frequency trend terms**

For the low-frequency trend series, its daily historical data is combined with the monthly GECON index [51], the MIDAS approach is employed to accomplish frequency alignment of different frequency data [52], and then the mixed-frequency data are employed as the input vector. The MFCBA approach can effectively extract the interactive features between the data, fully capturing the intrinsic relationships within frequency-aligned mixed-frequency input vectors [11]. The inclusion of the monthly low-frequency variable can predict the daily low-frequency trend components more accurately. Furthermore, SSA is used to find the optimal parameter combination for MFCBA to improve the efficiency of parameter selection.

(2) **Forecasting high-frequency disturbance terms**

The high-frequency disturbance terms exhibit significant stochasticity, time-varying, and clustering. The GARCH model is a powerful tool for handling stochastic processes that possess time-variant and volatility clustering attributes, making it well-suited for forecasting high-frequency components. This paper conducts an ARCH effect test on high-frequency series. If there is an ARCH effect, construct a GARCH prediction model; otherwise, build a deep learning prediction model [8].

#### 3.5.3. Step 3: Ensemble of Mode Components Forecasting Results

In step 3, the forecasts of all mode components with different frequency characteristics are summed to derive the final crude oil prices forecast.

### 3.6. Forecast Evaluation Criteria and Statistical Tests

This paper employs three measures, root mean square error (*RMSE*), mean absolute error (*MAE*), and mean absolute percentage error (*MAPE*), to assess the forecasting accuracy of different models. Their definitions are listed below:

(22) $$RMSE=\sqrt{\frac{1}{n}\sum_{t=1}^{n}{\left[x\left(t\right)-\hat{x}\left(t\right)\right]}^{2}},$$

(23) $$MAE=\frac{1}{n}\sum_{t=1}^{n}|x\left(t\right)-\hat{x}\left(t\right)|,$$

(24) $$MAPE=\frac{1}{n}\sum_{t=1}^{n}\frac{|x\left(t\right)-\hat{x}\left(t\right)|}{x\left(t\right)},$$

where x(t) and $\hat{x}(t)$ are the actual and forecasted values of the crude oil price at time t, respectively.

This paper employs the Diebold–Mariano (DM) test and model confidence set (MCS) test to assess the statistical significance of the proposed model. The MCS test includes a group of tests within the set $M_{0}$ to eliminate models with low predictive power. The set ${\hat{M}}_{\alpha }^{*}$ comprises the optimal predictive models at a confidence level of 1−α. For a given model $k\;\left(k\in M_{0}\right)$, the model belongs to the set ${\hat{M}}_{\alpha }^{*}$ if the p-value of its MCS test is larger than α. A larger *p*-value indicates better predictions [53]. Four indicators are selected as criteria: MSE, MAE, heteroskedasticity-adjusted MSE (HMSE), and heteroskedasticity-adjusted MAE (HMAE). Two statistics, $T_{R}$ and $T_{max}$, are obtained by bootstrap with 5000 replications, and α is set to 0.25. Additionally, the null hypothesis $H_{0}$ of the DM test is that the target model A and the benchmark model B possess identical forecasting capacities. $H_{0}$ is rejected at p<0.05, meaning they have different effects. Furthermore, if the statistic is positive, model B is better than model A [54].

## 4. Empirical Study

### 4.1. Data Description

**Figure 3:**
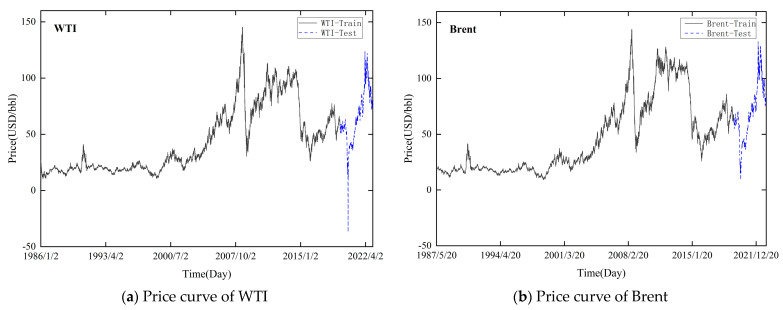
Daily international crude oil price curve.

For the empirical research, we employ WTI and Brent crude oil spot prices obtained from the US Energy Information Administration. Figure 3 shows the price curves of WTI and Brent. For WTI, the total dataset spans from 2 January 1986, to 21 February 2023, excluding the closing spot price on weekend trading days, with 9357 samples. The total dataset for Brent covers the period from 20 May 1987, to 21 February 2023, with 9077 samples. The sample set is divided into the training and test sets, accounting for 90% and 10%, respectively. Table 1 presents the descriptive statistics for WTI and Brent.

When forecasting low-frequency trend terms, we incorporate the monthly GECON index developed by Baumeister et al. (2022) [51], which reflects the current state of the global economy. This index is derived from 16 indicators related to real economic activity, such as commodity prices and financial indicators. The data can be accessed at https://sites.google.com/site/cjsbaumeister/datasets, accessed on 11 March 2023.

**Figure 4:**
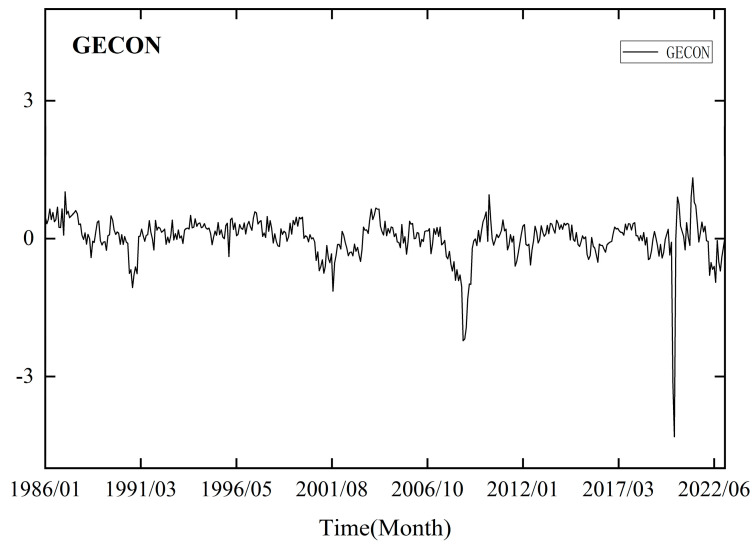
Global economic conditions index curve.

Figure 4 shows the time trend of GECON. Comparing the time series curve of GECON and crude oil prices, it is evident that there were significant declines around 2001, 2008, and 2020, indicating a high degree of correlation. Furthermore, previous studies have established a close relationship between global economic activity and crude oil prices through modeling, suggesting that GECON can serve as an important indicator for predicting crude oil prices [2,3,4]. Therefore, we further enhance the model’s predictive ability by performing deep learning network modeling based on monthly and daily mixed-frequency data.

Following the practice of Girardin and Joyeux (2013) [52], Xu et al. (2019) [55], and Cai et al. (2020) [11], the MIDAS method is used to deal with the mixed-frequency problem. The data sampled at different frequencies are frequency aligned, the lagged observations are treated as their variables (which can be considered as a feature engineering process from the perspective of machine learning), and then the mixed-frequency information set is input into the neural network for feature learning. In addition, to avoid the influence of data dimension inconsistency on model training, this study carried out the normalization of data when constructing the mixed-frequency deep learning model.

### 4.2. Decomposition of Crude Oil Prices

**Figure 5:**
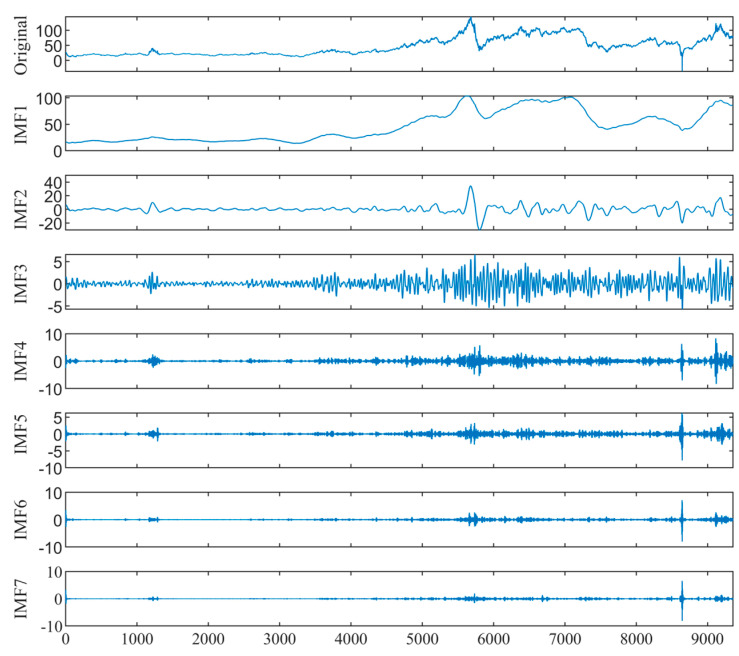
The KL-VMD decomposition results of WTI crude oil prices.

**Figure 6:**
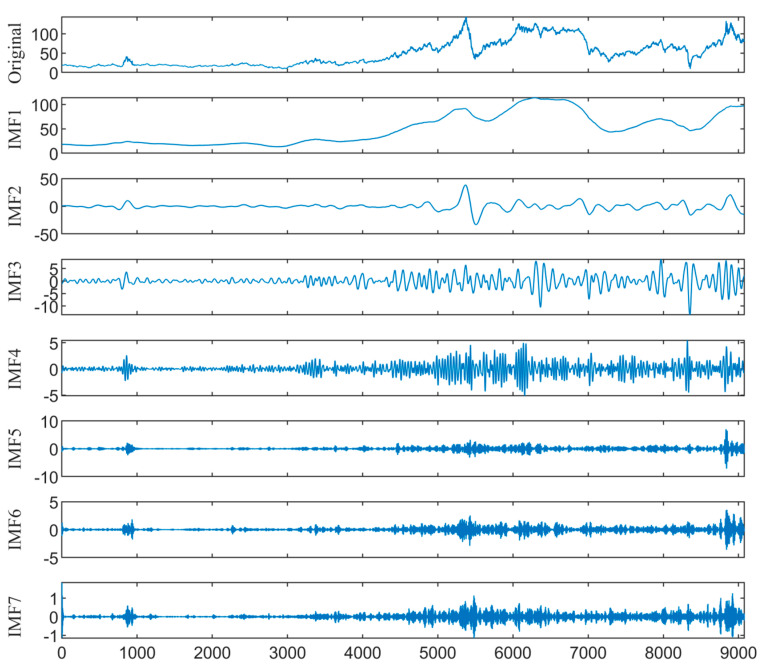
The KL-VMD decomposition results of Brent crude oil prices.

The KL-VMD method is employed to decompose the WTI and Brent prices. The K value and penalty factor α of decomposition are 7 and 900 for WTI, and 7 and 2100 for Brent, respectively. Figure 5 and Figure 6 show the KL-VMD results of WTI and Brent, with mode component frequencies from low to high. Noticeable cyclical variations occur in the low-frequency components, reflecting the long-term trend. However, the high-frequency components are characterized by stochasticity and volatility clustering. Compared with the original data, the decomposed mode components exhibit a simplified structure and high regularity, which improves the fitting and forecasting abilities.

### 4.3. Recognition of Mode Component Characteristics

We employ the FE algorithm to identify the frequency characteristics of the mode components, with parameter settings $m=2,n=2,r=0.15\sigma_{sd}$. Based on the FE values to classify the mode components, Table 2 presents the FE values and classification results of all components. Specifically, the FE values of mode components 1–2 of WTI and Brent are less than λ=0.05; thus, they are identified as low-frequency trend terms. Conversely, the FE values of mode components 3–7 are greater than λ, and are recognized as high-frequency disturbance terms.

### 4.4. Model Selection and Parameter Description

We prove the superiority of the proposed model through a comparative analysis of different models. Table 3 presents the abbreviations used for the models.

(1) We evaluate the forecasting performance between decomposition–integration models and the single model by introducing Model 1 (LSTM) for comparative analysis.
(2) Compared with Model 1, Models 2–4 all employ VMD for price decomposition before forecasting by LSTM. Specifically, Model 3 is optimized based on Model 2 using KL-VMD. Furthermore, Model 4 optimizes the parameters of LSTM using SSA, building on the enhancement in Model 3. Three questions can be evaluated by comparing Models 1–4: whether VMD can improve prediction accuracy, whether KL-VMD is better than VMD, and whether SSA-LSTM is better than LSTM.
(3) The remaining models adopt the decomposition–integration framework, employing the FE algorithm to divide mode components, forecasting high-frequency disturbances by GARCH, and optimizing deep learning parameters by SSA. Their distinctions are as follows: KL-VMD or EMD can be used in the mode decomposition stage. Perform pairwise comparisons between Models 5 and 7, 6 and 9, and 7 and 10. These comparisons can evaluate whether the decomposition performance of KL-VMD is superior to EMD. Next, LSTM, MFLSTM, and MFCBA can be used in the low-frequency component prediction stage. Sequentially comparing Models 5–7 and 8–10 can evaluate whether including mixed-frequency data can improve the prediction accuracy of LSTM and whether CBA has an advantage over LSTM.

**Figure 7:**
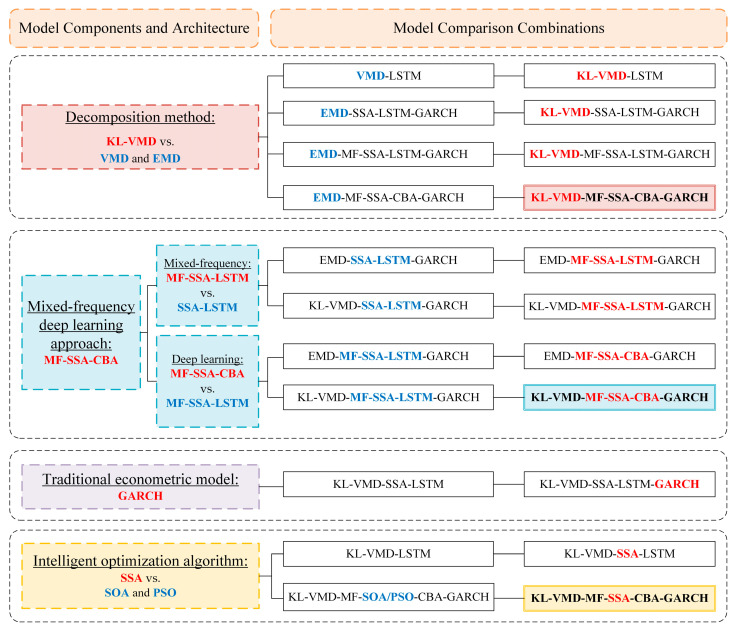
Model comparison combinations of each model component.

By selecting the aforementioned 10 models for comparison, this study establishes a series of model comparison combinations for each component within the model architecture. Figure 7 illustrates the detailed comparison combinations, primarily comparing the following four components of the proposed model:

(1) In the stage of decomposition, the decomposition effect of KL-VMD is compared with that of VMD and EMD.
(2) In the stage of low-frequency trend prediction using mixed-frequency deep learning approaches, MF-SSA-LSTM is compared with SSA-LSTM to determine whether the introduction of mixed-frequency data could improve prediction performance. Then, MF-SSA-CBA is compared with MF-SSA-LSTM to determine whether the specific mixed-frequency deep learning method adopted in this study is superior to LSTM.
(3) In the stage of high-frequency disturbance prediction using traditional econometric models, a comparison between KV-SL-G and KV-SL is conducted to verify whether the introduction of the GARCH model can enable the deep learning method and traditional econometric model to “perform their respective roles”, thereby enhancing predictive accuracy.
(4) Finally, as for the intelligent optimization algorithm SSA, KL-VMD-LSTM is compared with KL-VMD-SSA-LSTM to determine whether SSA can improve the prediction accuracy of deep learning models. In addition, in the discussion section, the prediction effect of models using SSA, SOA, and PSO for parameter optimization is compared to further verify the superiority of SSA.

Regarding the parameters of MFSCBA, we specify the number of sparrows and the maximum iterations in SSA as 10 and optimize the number of hidden units, iterations, and batch sizes in MFCBA with SSA, and the parameter search ranges are $\left[50,\;500\right],\;[50,\;500]$, and $\left[100,\;1000\right]$, respectively. Additionally, when the ARCH effect of the residuals is observed, we employ ARIMA-GARCH to model the mean and variance. The maximum lag order of each term is 3, and the Bayesian information criterion is used to make the selection. All calculations are implemented by Matlab R2022a and Python3.11 on 64-bit Windows 10.

### 4.5. Prediction Evaluation and Test Results

**Figure 8:**
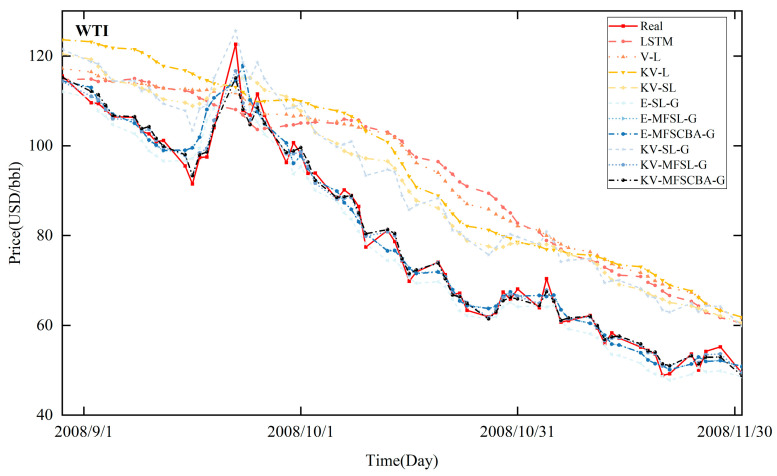
In-sample fitting results of each model for WTI crude oil prices.

**Figure 9:**
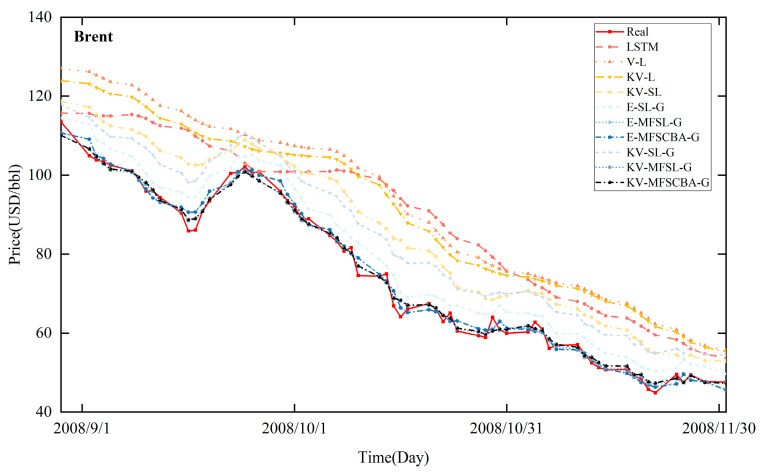
In-sample fitting results of each model for Brent crude oil prices.

**Figure 10:**
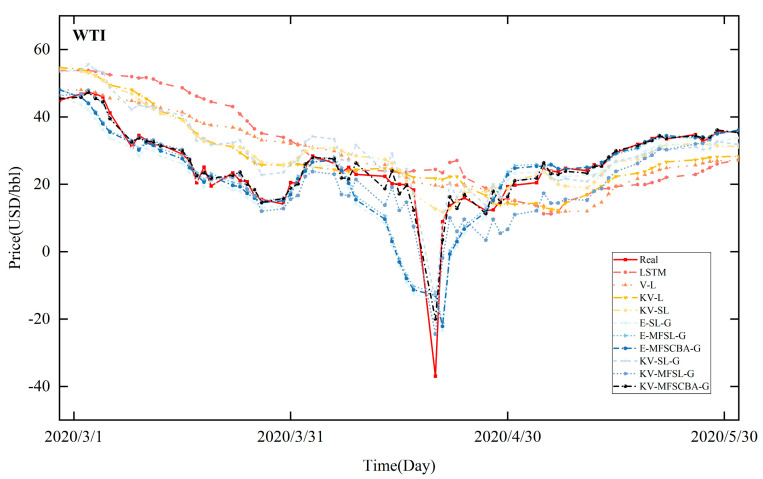
Out-of-sample forecasting results of each model for WTI crude oil prices.

**Figure 11:**
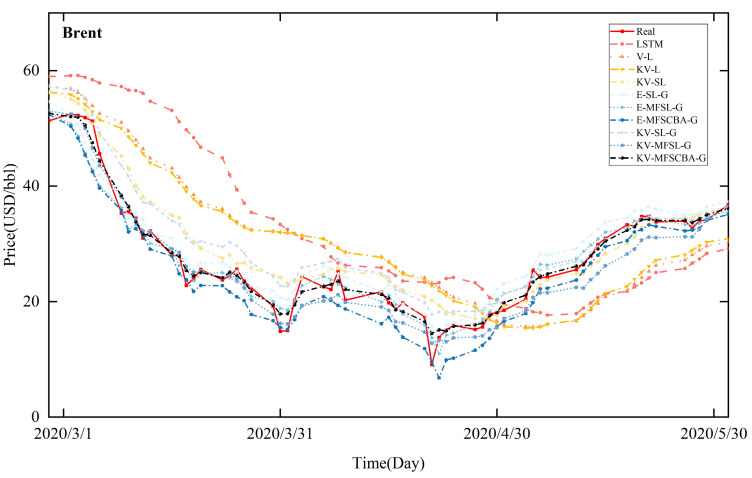
Out-of-sample forecasting results of each model for Brent crude oil prices.

Figure 8, Figure 9, Figure 10, and Figure 11 present the in-sample fitting and out-of-sample forecasting results of WTI and Brent, respectively. For clarity, only the three months with significant fluctuations are shown in each graph. Compared to alternative models, the KV-MFSCBA-G model has higher accuracy in values and directions fitted or predicted and more coherent upward or downward movement with the actual data.

To further assess the prediction accuracy of different methods, the in-sample fitting and out-of-sample forecasting errors of WTI and Brent under the three evaluation criteria are given in Table 4 and Table 5. Upon comparison, the following findings are made:

(1) In general, LSTM exhibits larger errors than all decomposition–integration models, proving the effectiveness of the decomposition–integration paradigm for oil price forecasting. Decomposition transforms the complicated price series into a simplified, stable, and regular structure, significantly enhancing forecasting accuracy.
(2) A comparison of the LSTM, V-L, KV-L, and KV-SL models reveals a progressive improvement in prediction accuracy, indicating that VMD contributes to improved accuracy, KL-VMD is better than VMD, and SSA-LSTM is better than LSTM. Thus, it proves the effectiveness of optimizing VMD and the deep learning approach by using KL divergence and the SSA intelligent optimization algorithm, respectively.
(3) Comparing the combinations of E-SL-G and KV-SL-G, E-MFSL-G and KV-MFSL-G, and E-MFSCBA-G and KV-MFSCBA-G, respectively, demonstrates that the KL-VMD method has a superior decomposition effect compared to EMD.
(4) A comparison of KV-SL-G, KV-MFSL-G, and KV-MFSCBA-G shows that considering mixed-frequency data enhances the prediction accuracy of LSTM. Furthermore, MFCBA exhibits superior performance compared to MFLSTM. Comparing the E-SL-G, E-MFSL-G, and E-MFSCBA-G models can confirm these findings.
(5) It is worth pointing out that the prediction accuracy of KV-SL-G is higher than KV-SL, indicating that the FE algorithm is used to divide components into low and high frequencies, and then the GARCH model is introduced to model the high-frequency disturbance, which effectively combines the advantages of the traditional econometric model and deep learning approach, thus improving the prediction accuracy.

In general, the KV-MFSCBA-G model nearly achieves the optimal forecasting performance in both WTI and Brent empirical studies, proving its robustness. It gives full play to KL-VMD’s capacity for decomposition denoising, the MFCBA model’s strong forecasting ability for nonlinear price series, the GARCH model’s good portrayal ability for volatility clustering, and SSA’s ability to find the optimal parameters efficiently.

Table 6 shows the MCS test results. Only the KV-MFSCBA-G method is always in $M_{75\%}^{*}$, and most of its *p*-values are 1. This finding validates the strength and robustness of the novel method.

Table 7 presents the DM test results. When comparing LSTM as the target model to the benchmark models, all *p*-values are smaller than 0.1, and the statistics are greater than 0, indicating that decomposition–integration forecasting models are significantly superior to the single model. Furthermore, when the remaining models are the target models, and KV-MFSCBA-G is the benchmark model, all *p*-values are smaller than 0.1, and the statistics are positive, confirming that the KV-MFSCBA-G method has a more outstanding crude oil price forecasting performance than the others.

Overall, this section begins with the baseline LSTM model and conducts a longitudinal comparative analysis step-by-step. The results of error measures and statistical tests confirm the superiority of the proposed model and the rationality of the chosen model components and architecture.

## 5. Discussion

### 5.1. Further Comparison against Existing Models in the Literature

Section 4 focuses on the longitudinal comparison of models. Following this, Section 5 will proceed to conduct a horizontal comparison of the performance of the proposed model with existing models in the literature. The selected comparative models include traditional econometric models, such as ARIMA [15,16] and ARIMA-GARCH [21,22]; machine learning methods, such as ANN [24], ELM [28], SVM [23], and XGBoost [26]; and hybrid models, including EEMD-GRU [33], EEMD-LSTM [32], and VMD-SVM-ARMA [35].

Table 8 and Table 9 present the in-sample fitting and out-of-sample forecasting performance of these comparison models and the proposed KV-MFSCBA-G model, respectively. Additionally, to enhance the transparency of the model architecture, the prediction accuracy of MF-SSA-CBA (the overall prediction accuracy of the low-frequency part of KV-MFSCBA-G) has been included in the final row of the tables.

Through comparison of the forecasting results of the models for WTI and Brent, it is evident that the proposed KV-MFSCBA-G model exhibits the smallest RMSE, MAE, and MAPE values, indicating the highest prediction accuracy. Additionally, the following findings are made:

(1) The forecasting accuracy of ARIMA-GARCH exceeds that of ARIMA, indicating that due to the highly volatile and non-constant variance characteristics of crude oil prices, ARIMA-GARCH can more accurately capture its dynamic features, leading to improved forecasting accuracy.
(2) The prediction error obtained from forecasting the low-frequency part using MF-SSA-CBA is much smaller compared to that of traditional econometric models and machine learning models. This highlights the ability of MF-SSA-CBA to better capture the underlying multi-scale complex features, long short-term dependencies, and non-linear trends of the low-frequency trend components. Furthermore, during the model training and prediction processes, MF-SSA-CBA can adaptively focus on crucial information, demonstrating greater flexibility and generalization ability, thus improving the predictive performance of the model.
(3) Another finding is that SVM demonstrates the best predictive performance among the four machine learning models. Similarly, VMD-SVM-ARMA showed the highest predictive performance among the three decomposition ensemble models. However, the improvement in accuracy of VMD-SVM-ARMA compared to SVM was not significant. The analysis suggests that although VMD can extract multiscale features from data to help deep learning models better understand long-term dependencies, it may not provide SVM with additional useful information and may even introduce noise or redundant information.

Therefore, it is reasonable to explore appropriate deep learning models to enhance model prediction performance for the low-frequency components obtained by KL-VMD. Additionally, combining the mixed-frequency deep learning approach with the traditional econometric model can fully utilize their respective strengths. The MFSCBA method is effective in describing complex nonlinear features, and the GARCH model is capable of capturing the volatility clustering effect, thus resulting in improved prediction accuracy.

### 5.2. Comparison of SSA with Other Intelligent Optimization Algorithms

To demonstrate the superiority of selecting SSA to optimize the MFCBA model in this study, we compared it with intelligent optimization algorithms used in the literature for crude oil price forecasting, including SOA [33] and PSO [35]. The parameter optimization search ranges of SOA and PSO are kept consistent with SSA, and the population size and maximum number of iterations of these three optimization algorithms are uniformly set to 10. To differentiate between the optimization algorithms used in each model, we denote our proposed KV-MFSCBA-G model as KV-MF-SSA-CBA-G. The models that use SOA and PSO are denoted as KV-MF-SOA-CBA-G and KV-MF-PSO-CBA-G, respectively. These three models differ only in their parameter optimization algorithms used for the MFCBA model.

**Figure 12:**
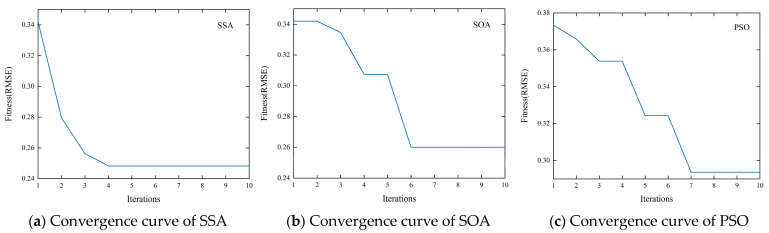
Convergence curves of different optimization algorithms.

From the convergence curves shown in Figure 12, taking WTI’s first low-frequency trend term as an example, although all three models reach convergence before 10 iterations, SSA converges faster and more efficiently with a smaller fitness function (RMSE).

Table 10 shows the in-sample and out-of-sample prediction performance of the three models for WTI and Brent. The model optimized by SSA can further improve prediction accuracy compared to SOA and PSO.

These results demonstrate that optimizing the deep learning model using SSA has certain benefits, including faster convergence speed, better global search capability, and less susceptibility to local optima, which effectively improves model prediction performance. Additionally, the program of the SSA had shorter running times in this study. This further validates the rationality of selecting SSA as the intelligent optimization algorithm component of the model architecture.

### 5.3. Economic Significance and Practical Application

Accurately forecasting the trend of international crude oil prices is crucial for ensuring global energy supply security and economic stability. It is also an important research focus in the field of global energy research. This study provides a scientifically precise forecast of international crude oil prices, which holds great economic implications and wide practical application. The findings can serve as a reference and basis for management decisions and risk control by market investors, policymakers, and market analysts.

Firstly, this study can assist investors in accurately analyzing and predicting trends in international crude oil prices. By combining the mixed-frequency deep learning approach, intelligent optimization algorithm, and traditional econometric model, the proposed model can capture the complex, nonlinear dynamic characteristics of crude oil prices. Furthermore, by introducing GECON data, the proposed model not only takes into account the internal dynamic factors of the crude oil market but also situates it within the macro context of the global economic environment, which results in more precise and dependable forecast results. As a result, this study can offer investors better insight into market price forecasts, enabling them to make better investment decisions, avoid market risks, and enhance investment returns.

Secondly, this study provides a reference basis for policymakers to formulate energy and monetary policies. Government agencies and regulatory bodies can utilize the proposed model to monitor and adjust the crude oil market in real time, promptly responding to changes in the global economic environment and market fluctuations.

Finally, this study is of great importance to oil-related enterprises when formulating production and investment strategies. In the face of uncertain crude oil prices, enterprises can use the proposed model to develop more effective strategies, which can help mitigate operational risks caused by market fluctuations and enhance competitiveness and profitability.

### 5.4. Future Directions

This study enhances the prediction performance of the model by incorporating monthly GECON data to construct the mixed-frequency deep learning model, and future research could expand the data sources for predicting crude oil prices by integrating more mixed-frequency data and multi-source heterogeneous data. Additionally, it would be a valuable research direction to introduce explainable artificial intelligence technology to open the black box of deep learning models and improve the interpretability and credibility of forecasting models.

## 6. Conclusions

Over the past few years, crude oil prices have fluctuated significantly. Therefore, we construct a novel KV-MFSCBA-G model for accurate forecasting, taking full advantage of the KL-VMD method’s noise reduction capability, the deep learning method’s superiority in forecasting nonlinear time series, the GARCH model’s strength in portraying volatility clustering, and the SSA’s efficient search speed and optimization ability. In addition, by combining the idea of mixed-frequency forecasting, we introduce the monthly exogenous economic variable to predict low-frequency trend terms from decomposition, improving the prediction accuracy. The empirical study and discussion in this paper encompass the longitudinal comparison starting from the baseline LSTM model; the horizontal comparison with existing models in the literature; and the comparison of optimization algorithms. The comparison of WTI and Brent crude oil price prediction results at these three levels consistently indicates that the KV-MFSCBA-G model proposed in this paper demonstrates superior prediction accuracy and robustness. It exhibits remarkable strengths in precisely forecasting nonlinear and highly volatile crude oil prices, validating the rationality behind the model’s selection of components and architecture. The main conclusions are as follows.

(1) The forecast error of LSTM is almost larger than that of all decomposition–integration models, verifying that the decomposition–integration paradigm is practical for crude oil price prediction. The decomposition effect of KL-VMD surpasses that of VMD and EMD, and it has a considerable advantage in improving model forecasting accuracy.
(2) The MFCBA and MFLSTM considering mixed-frequency data are more accurate than LSTM considering only historical crude oil prices, indicating that including mixed-frequency data enhances prediction accuracy. Moreover, MFCBA outperforms MFLSTM in forecasting, as validated under EMD and KL-VMD, illustrating that CBA incorporates the advantages of CNN, BiLSTM, and Attention, resulting in improved prediction accuracy compared to LSTM.
(3) Applying the FE algorithm for the frequency classification of components and using GARCH to forecast high-frequency disturbance components yields higher prediction accuracy than using the deep learning method for all components, highlighting the effectiveness of combining deep learning with traditional econometric models.
(4) SSA is used to optimize parameter combinations for LSTM, MFLSTM, and MFCBA. Deep learning models optimized by SSA demonstrate higher prediction accuracy than models with subjectively determined hyperparameters. Additionally, SSA exhibits faster convergence speed and superior computational efficiency compared to algorithms such as SOA and PSO, resulting in enhanced prediction accuracy.

In conclusion, this paper provides a novel method for forecasting international crude oil prices. Compared with other methods, the unique contributions and improvements of this study lie in (1) the combination of the mixed-frequency deep learning approach and traditional econometric models, (2) the introduction of monthly GECON data through the idea of MIDAS to improve forecast accuracy, and (3) the use of K-L divergence and intelligent optimization algorithm SSA to optimize the parameters. Additionally, potential drawbacks include the increased complexity of the model and longer runtime. The proposed model provides a scientifically accurate forecast of international crude oil prices and reveals that paying attention to global economic conditions can further enhance forecasting accuracy. Thus, this study can provide valuable guidance to market participants in shaping investment strategies and assist relevant departments in formulating policy decisions.

## Figures and Tables

**Table 1:** The summary of the crude oil price data set.

Oil	Data Set	Mean	Maximum	Minimum	Median	St. Dev.	Sample Size	Date Range
WTI	Full Set	46.1507	145.3100	−36.9800	36.1200	29.6021	9357	02/01/1986–21/02/2023
	Training Set	43.9452	145.3100	10.2500	30.3300	29.4187	8421	02/01/1986–29/05/2019
	Test Set	65.9924	123.6400	−36.9800	63.2750	23.1566	936	30/05/2019–21/02/2023
Brent	Full Set	48.6744	143.9500	9.1000	39.5400	32.8754	9077	20/05/1987–21/02/2023
	Training Set	46.2479	143.9500	9.1000	31.0500	32.7763	8169	20/05/1987–25/07/2019
	Test Set	70.5045	133.1800	9.1200	68.9100	24.6949	908	26/07/2019–21/02/2023

**Table 2:** Fuzzy entropy of each component and classification result.

IMFs	IMF_1_	IMF_2_	IMF_3_	IMF_4_	IMF_5_	IMF_6_	IMF_7_
Classification results of WTI	0.0021	0.0375	0.1796	0.3456	0.5217	0.6223	0.4038
	Low-frequency trend terms		High-frequency disturbance terms				
Classification results of Brent	0.0012	0.0224	0.0808	0.1961	0.3260	0.4320	0.3743
	Low-frequency trend terms		High-frequency disturbance terms				

**Table 3:** The abbreviations of the models.

Number	Model	Abbreviation
1	LSTM	LSTM
2	VMD-LSTM	V-L
3	KL-VMD-LSTM	KV-L
4	KL-VMD-SSA-LSTM	KV-SL
5	EMD-SSA-LSTM-GARCH	E-SL-G
6	EMD-MF-SSA-LSTM-GARCH	E-MFSL-G
7	EMD-MF-SSA-CBA-GARCH	E-MFSCBA-G
8	KL-VMD-SSA-LSTM-GARCH	KV-SL-G
9	KL-VMD-MF-SSA-LSTM-GARCH	KV-MFSL-G
**10**	**KL-VMD-MF-SSA-CBA-GARCH**	**KV-MFSCBA-G**

**Table 4:** Comparison of the in-sample fitting performance of the models.

Model	WTI			Brent		
	RMSE	MAE	MAPE	RMSE	MAE	MAPE
LSTM	3.8247	2.5213	0.0522	3.8406	2.6951	0.0485
V-L	3.3393	2.1063	0.0454	3.6266	2.2576	0.0425
KV-L	3.0785	1.7912	0.0390	3.2627	1.9598	0.0377
KV-SL	2.5179	1.5691	0.0356	1.8993	1.2114	0.0226
E-SL-G	2.0383	1.7089	0.0479	2.2085	1.8839	0.0641
E-MFSL-G	1.3553	0.9422	0.0236	1.2994	0.9308	0.0249
E-MFSCBA-G	0.7894	0.5247	0.0129	0.7848	0.5173	0.0121
KV-SL-G	2.3882	1.4894	0.0301	1.7395	1.1665	0.0294
KV-MFSL-G	1.5408	0.9213	0.0231	1.4588	0.8587	0.0206
**KV-MFSCBA-G**	**0.2389**	**0.1655**	**0.0043**	**0.1592**	**0.1181**	**0.0033**

**Table 5:** Comparison of the out-of-sample forecasting performance of the models.

Model	WTI			Brent		
	RMSE	MAE	MAPE	RMSE	MAE	MAPE
LSTM	6.3782	4.3695	0.0598	6.4632	4.5930	0.0605
V-L	5.1815	3.9522	0.0539	5.6364	4.2412	0.0564
KV-L	4.6433	3.3087	0.0444	5.1600	3.8526	0.0510
KV-SL	3.8657	2.8833	0.0390	2.8619	2.1653	0.0294
E-SL-G	3.6579	2.7479	0.0932	2.5545	2.1847	0.0340
E-MFSL-G	3.0711	1.8695	0.0675	2.4591	1.9903	0.0388
E-MFSCBA-G	2.6367	1.2176	0.0699	1.6703	1.2086	0.0246
KV-SL-G	3.4609	2.6784	0.0453	2.4274	1.7758	0.0275
KV-MFSL-G	1.9986	1.5114	**0.0279**	1.9576	1.5026	0.0266
**KV-MFSCBA-G**	**1.3871**	**0.5836**	0.0299	**0.6707**	**0.3714**	**0.0096**

**Table 6:** Model confidence set test results.

	Model	Loss Function							
		MSE		MAE		HMSE		HMAE	
		TR	Tmax	TR	Tmax	TR	Tmax	TR	Tmax
WTI	LSTM	0.0038	0.0036	0.0000	0.0000	0.3032 *	0.5256 *	0.0010	0.0032
	V-L	0.0008	0.0036	0.0000	0.0000	0.3186 *	0.5256 *	0.0002	0.0022
	KV-L	0.0020	0.0036	0.0000	0.0000	0.3544 *	0.5256 *	0.0002	0.0032
	KV-SL	0.0046	0.0242	0.0000	0.0000	0.3186 *	0.5256 *	0.0028	0.2200
	E-SL-G	0.0000	0.0426	0.0000	0.0000	0.2242	0.5256 *	0.0000	0.0208
	E-MFSL-G	0.1160	0.1028	0.0000	0.0026	0.4030 *	0.5256 *	0.0756	0.3412 *
	E-MFSCBA-G	0.1754	0.4704 *	0.0000	0.0066	0.5318 *	0.5502 *	0.0756	0.4558 *
	KV-SL-G	0.0148	0.1028	0.0000	0.0026	0.3544 *	0.5914 *	0.0056	0.3412 *
	KV-MFSL-G	0.1754	0.4704 *	0.0000	0.0066	1.0000 *	1.0000 *	0.0756	0.4558 *
	**KV-MFSCBA-G**	1.0000 *	1.0000 *	1.0000 *	1.0000 *	0.5318 *	0.5914 *	1.0000 *	1.0000 *
Brent	LSTM	0.0026	0.0024	0.0000	0.0000	0.1748	0.4322 *	0.0018	0.0020
	V-L	0.0030	0.0024	0.0000	0.0000	0.0442	0.4322 *	0.0000	0.0000
	KV-L	0.0016	0.0024	0.0000	0.0000	0.1578	0.4322 *	0.0006	0.0006
	KV-SL	0.0068	0.2802 *	0.0000	0.0098	0.0992	0.6354 *	0.0000	0.2044
	E-SL-G	0.0000	0.2802 *	0.0000	0.0002	0.0228	0.4322 *	0.0000	0.2044
	E-MFSL-G	0.0084	0.2802 *	0.0000	0.0098	0.1748	0.4322 *	0.0538	0.2044
	E-MFSCBA-G	0.0016	0.2802 *	0.0000	0.0326	0.1748	0.6354 *	0.0000	0.2044
	KV-SL-G	0.0084	0.2802 *	0.0000	0.0326	0.1748	0.6354 *	0.0030	0.2044
	KV-MFSL-G	0.0012	0.2802 *	0.0000	0.0326	0.1748	0.6354 *	0.0018	0.2044
	**KV-MFSCBA-G**	1.0000 *	1.0000 *	1.0000 *	1.0000 *	1.0000 *	1.0000 *	1.0000 *	1.0000 *

Note: This table reports the *p*-value of the forecasting model under the MCS test. * indicates that the *p*-value is greater than 0.25, which means the model is in ${\hat{M}}_{75\%}^{*}$. Bold numbers indicate the best model in that set of tests.

**Table 7:** Diebold–Mariano test results.

Target Model		Benchmark Model								
		V-L	KV-L	KV-SL	E-SL-G	E-MFSL-G	E-MFSCBA-G	KV-SL-G	KV-MFSL-G	KV-MFSCBA-G
WTI	LSTM	3.811 (0.000 *)	6.663 (0.000 *)	6.980 (0.000 *)	5.823 (0.000 *)	8.267 (0.000 *)	7.376 (0.000 *)	6.182 (0.000 *)	7.749 (0.000 *)	**8.163** (0.000 *)
	V-L		3.489 (0.000 *)	8.126 (0.000 *)	5.818 (0.000 *)	9.751 (0.000 *)	9.106 (0.000 *)	9.123 (0.000 *)	14.047 (0.000 *)	**14.940** (0.000 *)
	KV-L			5.939 (0.000 *)	3.381 (0.001 *)	8.415 (0.000 *)	6.478 (0.000 *)	4.687 (0.000 *)	8.373 (0.000 *)	**9.331** (0.000 *)
	KV-SL				0.738 (0.461)	4.516 (0.000 *)	4.137 (0.000 *)	2.619 (0.009 *)	8.583 (0.000 *)	**9.957** (0.000 *)
	E-SL-G					2.208 (0.027 *)	22.398 (0.000 *)	0.681 (0.496)	4.647 (0.000 *)	**5.840** (0.000 *)
	E-MFSL-G						1.557 (0.119)	−1.449 (0.147)	3.251 (0.001 *)	**4.869** (0.000 *)
	E-MFSCBA-G							−2.610 (0.009 *)	1.574 (0.115)	**2.767** (0.006 *)
	KV-SL-G								14.393 (0.000 *)	**14.685** (0.000 *)
	KV-MFSL-G									**6.150** (0.000 *)
Brent	LSTM	4.480 (0.000 *)	7.287 (0.000 *)	11.585 (0.000 *)	11.715 (0.000 *)	10.444 (0.000 *)	12.953 (0.000 *)	12.142 (0.000 *)	12.570 (0.000 *)	**13.565** (0.000 *)
	V-L		11.465 (0.000 *)	16.115 (0.000 *)	14.967 (0.000 *)	12.073 (0.000 *)	17.115 (0.000 *)	16.225 (0.000 *)	16.295 (0.000 *)	**18.089** (0.000 *)
	KV-L			14.705 (0.000 *)	13.960 (0.000 *)	10.682 (0.000 *)	16.496 (0.000 *)	15.116 (0.000 *)	15.580 (0.000 *)	**17.642** (0.000 *)
	KV-SL				3.683 (0.000 *)	−2.042 (0.041 *)	12.287 (0.000 *)	8.292 (0.000 *)	10.078 (0.000 *)	**18.048** (0.000 *)
	E-SL-G					−4.696 (0.000 *)	14.231 (0.000 *)	1.682 (0.093 *)	8.493 (0.000 *)	**20.277** (0.000 *)
	E-MFSL-G						9.776 (0.000 *)	5.009 (0.000 *)	8.597 (0.000 *)	**12.680** (0.000 *)
	E-MFSCBA-G							−9.598 (0.000 *)	−4.475 (0.000 *)	**10.674** (0.000 *)
	KV-SL-G								6.847 (0.000 *)	**18.666** (0.000 *)
	KV-MFSL-G									**19.697** (0.000 *)

Note: * indicates that the *p*-value is smaller than 0.1.

**Table 8:** Comparison of the in-sample fitting performance of the models.

Model	WTI			Brent		
	RMSE	MAE	MAPE	RMSE	MAE	MAPE
ARIMA	1.1999	0.7163	0.0174	1.1785	0.6848	0.0162
ARIMA-GARCH	0.5982	0.3131	0.0104	0.6645	0.2969	0.0098
ANN	4.2182	2.6212	0.0605	5.4959	3.1558	0.0795
ELM	4.7265	3.0056	0.0652	5.3405	3.5666	0.0728
SVM	1.3179	0.9029	0.0234	1.2725	0.8722	0.0213
XGBoost	3.8672	3.4005	0.0548	5.3797	4.8179	0.0540
EEMD-GRU	2.4999	1.0885	0.0386	2.2363	1.0015	0.0278
EEMD-LSTM	3.4170	1.8078	0.0330	5.2917	4.2329	0.0633
VMD-SVM-ARMA	2.0170	1.8255	0.0650	2.2847	2.0917	0.0732
**KV-MFSCBA-G**	**0.2389**	**0.1655**	**0.0043**	**0.1592**	**0.1181**	**0.0033**
MF-SSA-CBA	0.2370	0.1658	0.0042	0.1594	0.1182	0.0033

**Table 9:** Comparison of the out-of-sample forecasting performance of the models.

Model	WTI			Brent		
	RMSE	MAE	MAPE	RMSE	MAE	MAPE
ARIMA	3.1295	1.5220	0.0320	2.1476	1.4418	0.0234
ARIMA-GARCH	2.5792	0.6055	**0.0205**	0.7368	0.4326	0.0100
ANN	6.0856	4.1018	0.0565	6.5666	4.4029	0.0659
ELM	8.7667	5.2618	0.0726	8.5546	6.0619	0.0854
SVM	3.0416	1.7153	0.0376	2.4677	1.7098	0.0272
XGBoost	4.2813	2.8198	0.0431	3.7023	2.8438	0.0349
EEMD-GRU	8.2296	3.1935	0.0740	7.7162	2.6895	0.0522
EEMD-LSTM	5.2889	3.9293	0.0583	5.4670	4.0812	0.0568
VMD-SVM-ARMA	2.0425	1.7570	0.0293	2.4288	2.1373	0.0315
**KV-MFSCBA-G**	**1.3871**	**0.5836**	0.0299	**0.6707**	**0.3714**	**0.0096**
MF-SSA-CBA	1.3890	0.5921	0.0236	0.6753	0.3738	0.0080

**Table 10:** Forecasting performance of models based on different optimization algorithms.

**In-Sample**	**WTI**			**Brent**		
	**RMSE**	**MAE**	**MAPE**	**RMSE**	**MAE**	**MAPE**
**KV-MF-SSA-CBA-G**	**0.2389**	**0.1655**	**0.0043**	**0.1592**	**0.1181**	**0.0033**
KV-MF-SOA-CBA-G	0.2586	0.1856	0.0051	0.2345	0.1835	0.0060
KV-MF-PSO-CBA-G	0.2639	0.1987	0.0059	0.2521	0.1970	0.0065
**Out-of-Sample**	**WTI**			**Brent**		
	**RMSE**	**MAE**	**MAPE**	**RMSE**	**MAE**	**MAPE**
**KV-MF-SSA-CBA-G**	**1.3871**	**0.5836**	**0.0299**	**0.6707**	**0.3714**	**0.0096**
KV-MF-SOA-CBA-G	1.4178	0.5955	0.0309	0.7402	0.4615	0.0115
KV-MF-PSO-CBA-G	1.5016	0.6138	0.0345	0.8581	0.5109	0.0138

## Data Availability

Available by corresponding author upon request.
